# A Comparative Evaluation of Nalbuphine and Tramadol for the Control of Post-Spinal Anaesthesia Shivering

**DOI:** 10.7759/cureus.20481

**Published:** 2021-12-17

**Authors:** Sonalika Tudimilla, Chhaya Suryawanshi, Khalki SaravanKumar

**Affiliations:** 1 Anaesthesiology, Dr. D. Y. Patil Medical College, Hospital & Research Centre, Pimpri, Pune, Dr. D. Y. Patil Vidyapeeth, Pune, Pune, IND

**Keywords:** sedation, vomiting, nausea, tramadol, nalbuphine, post spinal anaesthesia shivering

## Abstract

Objectives

In this study, our primary aim was to compare the efficacy of and haemodynamic changes related to nalbuphine and tramadol when used for the control of post-spinal anaesthesia shivering, as per Wrench shivering grades. The secondary aim was to study the complications and adverse effects associated with the drugs.

Methodology

A total of 60 patients with the American Society of Anesthesiologists (ASA) physical status class I/II who were scheduled to undergo elective surgeries under spinal anaesthesia were divided into two groups of 30 each. Group N received intravenous nalbuphine 0.05 mg/kg and Group T received intravenous tramadol 1 mg/kg, two minutes after the patients started shivering after undergoing spinal anaesthesia. The anaesthesia technique was standardised for all the patients in the study. The shivering grade was measured using the Wrench shivering grade and the level of sedation was studied using the Ramsay Sedation Scale. Heart rate (HR), systolic blood pressure (SBP), diastolic blood pressure (DBP), and mean arterial pressure (MAP) were recorded. All the parameters were measured at the baseline and at one, two, five, 10, 15, and 30 minutes after administering the drug.

Results

Immediately after giving the drug, the time taken to control shivering was significantly lower in Group T: 3.633 minutes. However both the drugs controlled shivering effectively. There were no significant haemodynamic changes in both groups, probably due to the lower dosage of drugs used in our study. A different set of side effects were seen in each group. In Group N, out of 30 patients, five (16.67%) patients were sedated, four (13.33%) had hypotension, and two (6.67%) had bradycardia, whereas In Group T, out of 30 patients, five (16.67%) patients had nausea, four (13.33%) had nausea and vomiting, and two (16.67%) had dizziness following the administration of the drug. Respiratory depression or itching was not seen in any patients in either group.

Conclusion

Based on our findings, both Intravenous nalbuphine 0.05 mg/kg and intravenous tramadol 1 mg/kg are effective in treating patients with post-spinal anaesthesia shivering; however, the time taken to control shivering is lower with tramadol than nalbuphine. Both the drugs resulted in minimal haemodynamic changes and adverse effects.

## Introduction

Regional anaesthesia (spinal anaesthesia) is conventionally used as a frequent, safe, and approved anaesthetic procedure. Post-spinal anaesthesia shivering is a significantly distressing and frequent complication, reported in about 40-70% of the patients who undergo surgical procedures under regional anaesthesia [1-2].

Perioperative hypothermia is the chief cause of post-spinal anaesthesia shivering, which occurs due to anaesthetic-induced inhibition of thermo-genesis causing cutaneous vasodilatation (triggered by pain) and decreased threshold for stimulation of vasoconstriction [3]. This leads to the dissemination of body heat from the core from the torso (beneath the level of the block) to the periphery with subsequent rapid hypothermia during anaesthesia [3]. Excessive post-spinal shivering can cause increased O_2_ consumption and CO_2_ production. This can cause metabolic disturbances such as hypoxaemia, lactic acidosis, hypercarbia, and catecholamine release, impeding a smooth recovery from anaesthesia [4]. Shivering, being a frequent complication, can potentially have adverse effects in high-risk patients, especially cardiac patients with fixed cardiac output and pulmonary patients with existent internal intrapulmonary shunts or restricted respiratory reserve. It can also lead to raised intraocular pressure and intracranial tension [5,6].

Diversified approaches are available for the control of post-spinal anaesthesia shivering, including non-pharmacological and pharmacological methods. Non-pharmacological approaches utilising equipment to maintain body temperature are efficient but costly, and they lack feasibility; they include blankets, plastic sheets, surgical drapes, space blankets, and insulators, etc. The pharmacological methods include drugs like ketamine, tramadol, meperidine, pethidine, nefopam, and clonidine [7]. Unfortunately, there is no gold standard treatment for shivering because the use of all available medications is associated with multiple negative side effects.

Over the past few decades, a synthetic opioid, tramadol, has been frequently recommended and used to control shivering after spinal anaesthesia. The anti-shivering property of tramadol is mediated by the inhibition of the reuptake of serotonin, norepinephrine, and dopamine as well as its ability to facilitate 5-HT release [8-10]. Though it has opioid-like characteristics, it lacks significant naloxone reversibility. Nevertheless, it has numerous undesirable effects, most commonly nausea, vomiting, and dizziness.

Nalbuphine is a semisynthetic, mixed agonist-antagonist opioid that contains both antagonist and agonist characteristics. In the central nervous system, nalbuphine has a higher affinity for opioid receptors. In the hypothalamus, nalbuphine inhibits shivering. Because of the large density of alpha-2 adrenoreceptors in the hypothalamus, nalbuphine reduces the temperature regulation threshold for vasoconstriction and shivering. It has a minimal respiratory depressant effect and a low potential for abuse compared to other centrally acting opioids [1,11-13].

This study was undertaken to compare the efficacy as well as the haemodynamic and adverse effects of nalbuphine and tramadol when used for the control of post-spinal anaesthesia shivering.

## Materials and methods

This prospective randomised (computer-generated) double-blinded study was conducted after obtaining approval from the Institutional Ethics Sub-Committee (Research Protocol Number: IESC/PGS/2019/146). Written informed consent was obtained from the patients after providing them with the patient information sheet. The study was conducted among 60 patients of either sex with the American Society of Anesthesiologists (ASA) physical status class I/II who were scheduled to undergo elective surgeries like lower limb surgeries and percutaneous nephrolithotomy under spinal anaesthesia administered by the Department of Anaesthesia, Dr. D. Y. Patil Medical College, Hospital & Research centre, Pune from August 2019 to September 2021. Randomisation was done using a computer-generated number where patients were randomly allocated into two groups: Group N and Group T. We confined our investigation to 60 patients divided into two equal groups of 30 people. The sample size estimation was done based on a study titled "Comparison between Tramadol Hydrochloride and Nalbuphine Hydrochloride in the Treatment of Perioperative Shivering After Spinal Anesthesia" by Haque et al. in 2011 [14]. Assuming a success rate of 85%, with an acceptable error of 13% at a confidence interval of 95%, the sample size worked out to 29, which was rounded off to 30 in each group. The sample size was calculated using the WinPepi statistical package.

Inclusion criteria

Patients aged between 21 and 60 years of both genders with ASA I and II status who were scheduled for elective surgeries under spinal anaesthesia were included in our study.

Exclusion criteria

Patients aged less than 20 years and more than 60 years, patients with a history of uncontrolled comorbidities, cardiac, respiratory, renal, or hepatic diseases or those who had a history of allergy to any of the test drugs, patients with contraindications to spinal anaesthesia (coagulation disorder, infection at the puncture site, raised intracranial tension, preexisting neurological deficits in lower extremities or any spine deformity), and patients with initial body temperature >38°C or <36°C were excluded from our study.

On the day before the scheduled surgery, a thorough pre-anaesthetic evaluation was done for all the patients. Patients were explained about the study procedure. All patients were kept nil by mouth for at least six hours prior to surgery. On the day of surgery, in the preoperative room, intravenous access was secured with a 20-gauge cannula, and patients were preloaded with 15-ml/kg Ringer’s lactate fluid and maintained on intravenous fluids throughout the surgery. In the operation room, minimum mandatory monitoring was carried out using a multiparameter monitor. Continuous monitoring of heart rate (HR), blood pressure (BP), peripheral oxygen saturation (SpO_2_), ECG, and axillary temperature monitoring was carried out throughout the procedure.

Under all aseptic precautions, and with patients in the sitting position, spinal anaesthesia was given at L3-L4 intervertebral space using 26-G Quincke Babcock spinal needle; after confirming free and clear flow of cerebrospinal fluid, 0.5% bupivacaine heavy (3/3.5 ml) was given to achieve the desired level according to the surgical procedure. An optimum temperature of around 22-24 °C was maintained in the operation theatres, and patients were covered with drapes but not actively warmed. Intravenous fluids and drugs were administered at room temperature.

After the induction of spinal anaesthesia, patients were observed for any occurrence of shivering until the postoperative period. Shivering was graded according to the following system (Table 1) [15].

**Table 1:** Wrench shivering grade

Grade	Muscle groups involved
0	No shivering
1	One or more of the following: piloerection, peripheral vasoconstriction, peripheral cyanosis, but without visible muscle activity
2	Visible muscle activity confined to one muscle group
3	Visible muscle activity in more than one muscle group
4	Gross muscle activity involving the whole body

Patients who developed either Grade 3 or 4 shivering were included in the study. Injection tramadol 1 mg/kg or injection nalbuphine 0.05 mg/kg were diluted to a volume of 10 ml in a 10-ml syringe. And the syringe was assigned as coded syringes as per the randomisation list by an anaesthesiologist who was unaware of the group allocation. This was then administered to the patient as a slow intravenous injection over a period of 10 minutes. The attending anaesthesiologist recorded in minutes the time of the onset of shivering after spinal anaesthesia, the time of the administration of the test drug, and the time to the cessation of shivering.

Shivering control was defined as "complete" when the shivering grade declined to 0; "incomplete" when the scores decreased but the shivering did not stop completely; and "failed" if no change in scores were observed. All the parameters were recorded at zero, one, two, five, 10, 15, and 30 minutes. In case there was a recurrence of shivering, patients were treated with an additional dose of intravenous tramadol (0.5 mg/kg) or intravenous nalbuphine (0.025 mg/kg IV), as per the respective groups, and/or active warming measures using convection heaters or infusing moderately warm intravenous fluids. Adverse effects were recorded, treated, and monitored.

Sedation was assessed as per the Modified Ramsay Sedation Scale (Table 2) [16].

**Table 2:** Modified Ramsay Sedation Scale

Score	Response
1	The patient is anxious or agitated or both
2	The patient is cooperative, oriented, and tranquil
3	The patient responds to commands only
4	A brisk response to a light glabellar tap
5	A sluggish response to a light glabellar tap
6	No response

A sedation score of more than 3 was termed sedation as a side effect.

Statistical analysis

The data were collected, compiled, and tabulated. The statistical analysis was done using a parametric test and the final interpretation was based on Z-test (standard normal variant) with a 95% level of significance. Quantitative data were analysed using the Student's t-test, and qualitative data were analysed with the chi-square test.

## Results

The demographic characteristics, ASA grade, duration of surgery, duration of spinal anaesthesia, and the onset of shivering were comparable between the two groups (Table 3).

**Table 3:** Demographic characteristics, ASA class, duration of surgery, spinal anaesthesia, and the onset of shivering in both groups BMI: body mass index; ASA: American Society of Anesthesiologists; SD: standard deviation

Parameter	Group N (nalbuphine) (n=30)	Group T (tramadol) (n=30)	P-value
Age (years), mean ± SD	38.466 ± 9.27	39.433 ± 10.99	0.714
Gender distribution (male:female)	14:16	17:13	-
BMI (kg/m^2^), mean ± SD	24.33 ± 4.02	24.68 ± 3.6	0.608
ASA I/II	17/13	16/14	-
Duration of surgery (minutes), mean ± SD	85.2 ± 11.59	90.96 ± 19.89	0.175
Duration of spinal anaesthesia (minutes), mean ± SD	232.33 ± 18.44	218.26 ± 19.48	0.06
The onset of shivering (minutes), mean ± SD	29.93 ± 4.20	30.77 ± 6.35	0.09

**Figure 1:**
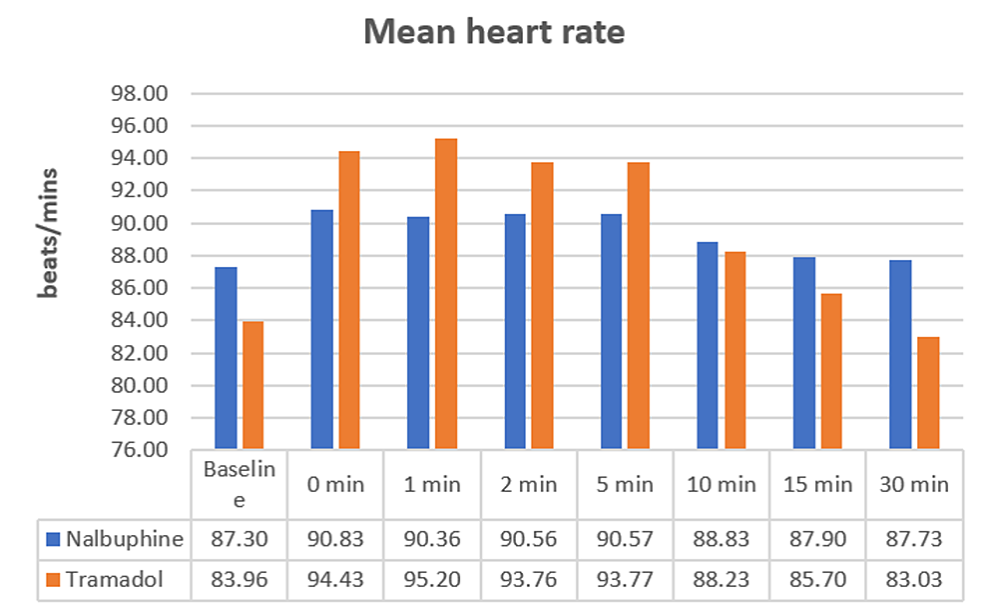
Mean heart rate

The HR was comparable in both groups and was not statistically significant (Figure 1).

The systolic blood pressure (SBP), diastolic blood pressure (DBP), and mean arterial pressure (MAP) were comparable in both groups and were not statistically significant (Table 4).

**Table 4:** Comparison of SBP, DBP, and MAP between groups SBP: systolic blood pressure; DBP: diastolic blood pressure; MAP: mean arterial pressure; N: nalbuphine; T: tramadol; SD: standard deviation

Time	SBP (mmHg)			DBP (mmHg)			MAP (mmHg)		
	N	T	P-value	N	T	P-value	N	T	P-value
	Mean ± SD	Mean ± SD		Mean ± SD	Mean ± SD		Mean ± SD	Mean ± SD	
Baseline	117.56 ± 11.49	112.8 ± 11.57	0.115	77.37 + 7.08	77.87 + 9.12	0.813	90.77 + 8.34	89.51 + 9.43	0.89
Zero minute after giving the drug	112.83 ± 10.43	114.16 ± 10.17	0.618	74.70 + 6.99	77.47 + 9.47	0.203	87.41 + 7.67	89.70 + 9.19	0.53
One minute after giving the drug	111.36 ± 10.60	116.4 ± 9.56	0.058	73.57 + 7.09	77.27 + 9.61	0.095	86.17 + 7.67	90.31 + 9.05	0.27
Two minutes after giving the drug	109.96 ± 11.093	117.2 ± 9.66	<0.01	73.33 + 7.16	77.93 + 9.41	0.052	85.54 + 7.58	91.02 + 9.03	0.10
Five minutes after giving the drug	109.43 ± 11.23	116.46 ± 9.49	0.011	72.53 + 7.08	78.80 + 8.90	0.065	84.83 + 7.59	91.36 + 8.69	0.06
10 minutes after giving the drug	109.33 ± 10.74	115.3 ± 9.40	0.026	72.63 + 7.12	77.87 + 8.97	0.120	84.87 + 7.45	90.34 + 8.64	0.09
15 minutes after giving the drug	109.76 ± 10.41	113.3 ± 9.64	0.178	73.03 + 7.12	77.57 + 9.73	0.06	85.28 + 7.36	89.48 + 9.14	0.13
30 minutes after giving the drug	109.9 ± 10.72	112.26 ± 9.87	0.378	73.53 + 6.84	76.50 + 9.49	0.17	85.66 + 7.34	88.42 + 8.95	0.33

**Figure 2:**
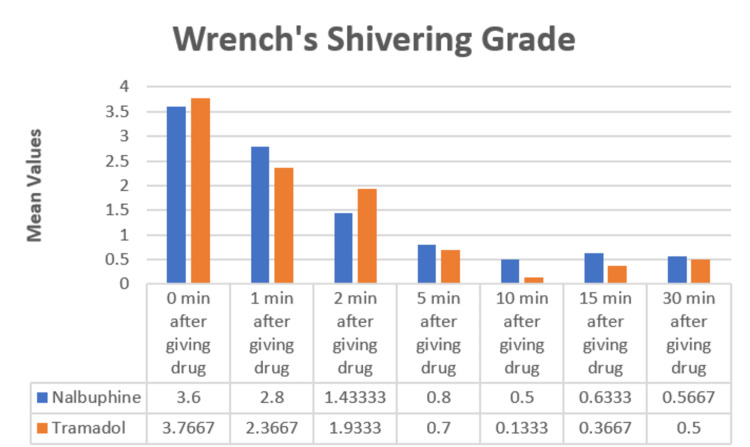
Bar diagram comparing Wrench shivering grade between groups

The Wrench shivering grade was also comparable in both groups (Figure 2).

The time taken to control shivering was comparable in both groups and was statistically significant (p=0.02) (Table 5).

**Table 5:** Time taken to control shivering SD: standard deviation

Parameter	Group N (n=30)	Group T (n=30)	P-value
	Mean ± SD	Mean ± SD	
Time taken to control shivering (minutes)	4.692 ± 1.64	3.633 ± 1.572	0.02

**Figure 3:**
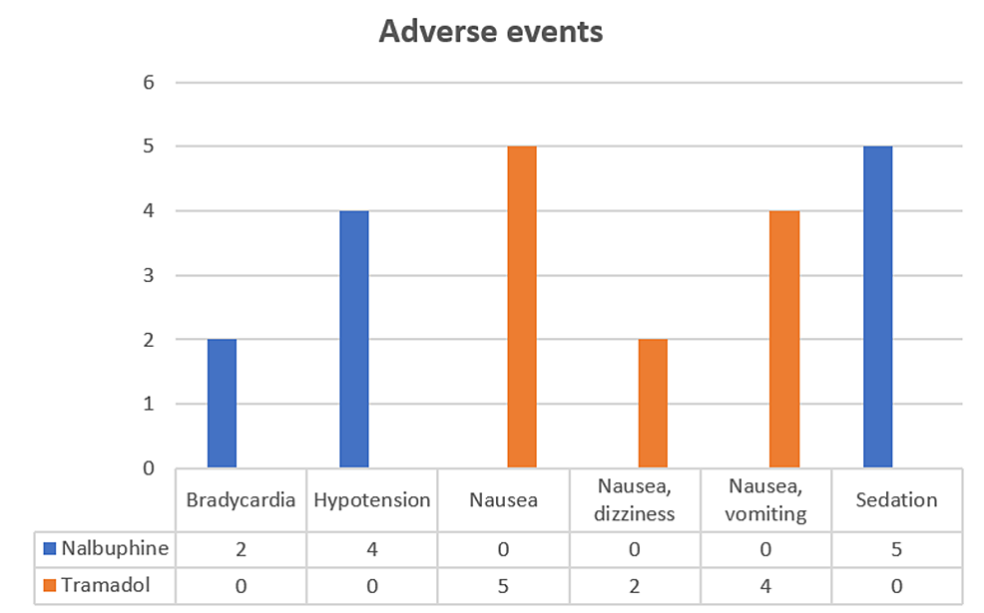
Comparison of adverse events between the groups

Adverse events were noted in a few patients, which were minor and treatable (Figure 3).

The Ramsay Sedation Scale score was comparable in both groups and was statistically insignificant (Table 6).

**Table 6:** Comparison of Ramsay Sedation Scale score between the groups SD: standard deviation

Parameter	Group N (n=30)	Group T (n=30)	P-value
	Mean ± SD	Mean ± SD	
Ramsay Sedation Scale score	2.3 ± 0.70	1.1 ± 0.3	0.06

## Discussion

Spinal anaesthesia is the most common anaesthetic technique used for lower abdominal and lower limb surgeries. Its rapid onset of action and association with postoperative analgesia makes it a very popular technique. However, as with all anaesthetic methods, spinal anaesthesia is also associated with various side effects.

Shivering is an involuntary muscular activity experienced by almost 40-50% of patients undergoing surgeries under spinal anaesthesia [1-2]. Shivering is a protective mechanism against hypothermia. Vigorous involuntary muscular activity generates heat in the body in response to hypothermia. It is a very discomforting sensation to a patient. In addition, shivering causes an increase in oxygen demand up to six times the normal requirement. This causes a mismatch between oxygen demand and supply, which can lead to side effects like hypoxaemia, hypercarbia, and lactic acidosis.

Even though the mechanism of shivering under regional anaesthesia is not fully understood, it appears to be mediated by norepinephrine, dopamine, neuropeptides, and 5-hydroxytryptamine as a response to hypothermia. Hypothermia occurs after spinal anaesthesia as a result of peripheral vasodilation of the lower part of the body causing heat loss, which is not compensated for by metabolic heat production of the body. Shivering can be controlled intraoperatively by various methods. Unfortunately, all the drugs have some kind of side effect and the perfect drug is yet to be discovered. And studies are being performed to find the best possible drug to control the shivering.

Nalbuphine is a semisynthetic, mixed agonist-antagonist opioid that has characteristics of µ-antagonist and ƙ-agonist activities. It has a high affinity for opioid receptors in the central nervous system. A clinically important contribution of k receptors is supported by the observation that meperidine, which is a µ and ƙ receptor agonist, reduces the intensity of cold-induced shivering even in the presence of moderate doses of nalbuphine. When compared to other opioids, it has a lower potential to impair respiratory function. Intravenous nalbuphine in the dose of 0.05 mg/kg was selected for our study.

Tramadol is a narcotic analgesic that works by acting on the central nervous system by inhibiting monoamine reuptake and appears to affect pain transmission by acting on opioid receptors. The racemate's analgesic efficacy and tolerance profile are improved by the complementary and synergistic activities of the two enantiomers. Tramadol interacts with μ, δ, and κ receptors where it purely exhibits agonistic activity. Tramadol's anti-shivering effect is most likely mediated by its opioid, serotonergic, and noradrenergic activities or both. Intravenous tramadol in the dose of 1 mg/kg was selected for our study.

The objective of our study was to compare the relative efficacy of intravenous tramadol 1 mg/kg with that of intravenous nalbuphine 0.05 mg/kg for the control of post-spinal anaesthesia shivering. Haemodynamic changes along with adverse effects like bradycardia, hypotension, nausea, vomiting, and others were also observed.

ASA grade 1 and 2 patients of both sexes of age group 21-60 years who were willing to undergo surgery under spinal anaesthesia were considered for our study. They were randomised into two equal groups. Each group included 30 patients. Computer-generated allocations were used after applying strict inclusion and exclusion criteria. Group N: this group received injection nalbuphine 0.05 mg/kg intravenously diluted in 10-ml normal saline, after the onset of shivering. Group T: this group received injection tramadol 1 mg/kg intravenously diluted in 10-ml normal saline, after the onset of shivering.

Demographic profile

In our study, the differences in gender, mean age, BMI, and ASA grading between Group N and Group T were not statistically significant (p>0.05) (Table 3).

Duration of surgery, duration of spinal anaesthesia, and time of onset of shivering

The duration of surgery, duration of spinal anaesthesia, and the time of onset of shivering were recorded and statistically analysed in both study groups. The difference between the groups was not statistically significant, thereby indicating that these parameters are not influenced by nalbuphine or tramadol (Table 3).

Wrench shivering grade

The shivering grades were subjected to statistical analysis and the difference was found to be statistically not significant at different intervals (p>0.05). Shukla et al., in 2011, compared the effect of intravenous clonidine 0.5 mcg/kg (Group C) and intravenous tramadol 0.5 mg/kg (Group T) on shivering and observed that the severity of shivering grade in Group T was 2.9 ± 1.2 while that in Group C was 3.1 ± 0.7 [17]. Nirala et al., in 2020, conducted a study comparing tramadol (1 mg/kg) (Group T) and nalbuphine (0.06 mg/kg) (Group N) for shivering, and a Wrench shivering grade of 3 was observed in 29 patients and grade 4 in 16 patients in group T, whereas in group N, grade 3 was found in 31 patients and grade four in 14 patients (p>0.05), which was not statistically significant [18]. They observed that both nalbuphine and tramadol had anti-shivering properties (Figure 2).

Time taken for the control of shivering

The mean value of time taken for control of shivering in the nalbuphine group was 4.692 ± 1.643 minutes and it was 3.633 ± 1.572 minutes in the tramadol group, and this difference was statistically significant (p=0.02). Hence, shivering was controlled significantly earlier in the tramadol group (Table 5). Reddy et al., in 2011, compared tramadol (50 mg) (Group T) and clonidine (50 mcg) (Group C) for shivering, and the time taken to control shivering in Group T was 2.2 ± 0.41 minutes with a response rate of 95.56%, whereas in Group C, it was 3.17 ± 0.03 minutes with a response rate of 86.67%. They observed that tramadol was more effective than clonidine in controlling shivering [19]. In the above-mentioned study by Nirala et al., the time taken for cessation of shivering in Group T was 4.84 ± 1.23 minutes, while it was 3.84 ± 1.23 minutes Group N, which led them to conclude that the time taken to control shivering was significantly lower in Group N when compared to Group T [18]. 

In our study, lower time was required to control shivering with tramadol than nalbuphine when compared with the mentioned studies. Our study results are consistent with that of a few studies.

Control of shivering

In our study, it was noted that shivering was not controlled in four patients in Group N and three patients in Group T; 25 patients (83.3%) had effective control in Group T whereas 23 patients (76.67%) had effective control in Group N; three patients (10%) and two patients (6.67%) had a partial control in both Groups T and N. Hence, the difference between the groups was not statistically significant based on the efficacy of drugs in suppressing the shivering. There was no recurrence of shivering in any of our study groups. Taneja et al., in 2019, conducted a study on tramadol (0.25 mg/kg) (Group 2) and nalbuphine (0.28 mg/kg) (Group 1) and observed an 85% response rate in Group 2 and 90% response rate in Group 1, with a 25% recurrence rate in Group 2 and 20% recurrence rate in Group 2. They concluded that nalbuphine is slightly more efficacious than tramadol [20]. Our study findings are consistent with a few studies with tramadol being more effective in controlling shivering, probably owing to the use of a higher dose when compared to other studies.

Haemodynamic parameters

In our study, there was no appreciable change in mean HR, SBP, DBP, MAP, and oxygen saturation. It was noted that there were no significant differences in all these parameters at all intervals of time. However, there was a gradual fall in BP subsequent to the administration of drugs in both groups when compared to the baseline, but this did not reach statistical significance (p>0.05) (Figure 1) (Table 4).

Adverse events

A different set of side effects was seen in each group. In Group N, out of 30 patients, five (16.67%) patients were sedated, four (13.33%) had hypotension, and two (6.67%) had bradycardia, whereas, in Group T, none of the patients had sedation, hypotension, or bradycardia. In Group T, out of 30 patients, five (16.67%) patients had nausea, four (13.33%) had nausea and vomiting, and two (16.67%) had dizziness following the administration of the drug. In group N, none of the patients experienced nausea or vomiting. Respiratory depression or itching was not seen in either of the group (Figure 3). Kyokong et al., in 2007, conducted a study to compare the efficacy of tramadol and nalbuphine and found that only 1.4% of patients had severe nausea in the tramadol group and 4.3% of patients were sedated in the nalbuphine group [13]. However, these side effects were few, minor, and treatable.

## Conclusions

Based on our findings, both intravenous nalbuphine 0.05 mg/kg and tramadol 1 mg/kg are effective in treating patients with post-spinal anaesthesia shivering. The time taken to control shivering is lower with tramadol than nalbuphine. Tramadol causes substantially greater nausea and vomiting, whereas nalbuphine causes considerable sedation. However, both the drugs led to minimal haemodynamic changes and negligible side effects, which were all minor and treatable. Further studies are needed for a better comprehension regarding the etiology and management of post-spinal anesthesia shivering.
